# A Rapid Review of Burns First Aid Guidelines: Is There Consistency Across International Guidelines?

**DOI:** 10.7759/cureus.15779

**Published:** 2021-06-20

**Authors:** Michael McLure, Finlay Macneil, Fiona M Wood, Leila Cuttle, Kathryn Eastwood, Janet Bray, Lincoln M Tracy

**Affiliations:** 1 Department of Epidemiology and Preventive Medicine, Monash University, Melbourne, AUS; 2 Department of Urology, Gosford District Hospital and Gosford Private Hospital, Gosford, AUS; 3 Faculty of Medicine, University of Newcastle, Newcastle, AUS; 4 State Adult Burn Unit, Fiona Stanley Hospital, Murdoch, AUS; 5 Burn Injury Research Unit, University of Western Australia, Perth, AUS; 6 School of Biomedical Science, Queensland University of Technology, South Brisbane, AUS; 7 Institute of Health and Biomedical Innovation, Queensland University of Technology, South Brisbane, AUS; 8 Children's Health Research Centre, Queensland University of Technology, South Brisbane, AUS; 9 Prehospital, Resuscitation and Emergency Care Research Unit, Curtin University, Bentley, AUS

**Keywords:** international guidelines, rapid review, burn first aid, guideline, burns and scalds

## Abstract

We conducted a rapid review of current international and Australian/New Zealand guidelines on first aid for burns to identify any critical variation and any recent major changes in the literature that would warrant a significant change to current recommendations. A search was conducted to identify Australian/New Zealand and international first aid guidelines for burn care using guideline databases, and we compared key recommendations from each guideline relating to burns first aid. A literature search of relevant databases (Medline, Embase, Cochrane Database of Systematic Reviews, PROSPERO international register of systematic reviews, and ClinicalTrials.gov databases) was conducted to identify existing and in-progress research published on the topic of first aid for burn injuries. Seven guidelines were identified from the Australia/New Zealand region, and 11 international guidelines were identified from the United States of America and Europe. All Australian and New Zealand guidelines recommended a cooling duration of 20 minutes and made some mention of when to refer a burn for medical evaluation, while international guidelines saw cooling duration variation, a number of guidelines failed to mention referral criteria. The review of published systematic reviews and clinical trials revealed a lack of new evidence in the last six years. Our rapid review identified key variation between first aid guidelines for burns that would benefit from the development of an international consensus on management. We identified no new significant evidence that would alter guideline recommendations and did not identify any upcoming reviews or clinical trials on this subject.

## Introduction and background

The World Health Organization (WHO) defines burns as “an injury to the skin or other organic tissue primarily caused by heat or due to radiation, radioactivity, electricity, friction or chemicals” [1]. Burns are a preventable but potentially life-altering injury that can have considerable impacts on a person’s health and quality of life and can also lead to death [2-5]. For people severely affected by burns, their injuries can disrupt all aspects of their physical, emotional, and financial well-being [2], as many patients require prolonged and often recurrent periods of surgical, medical, and psychological rehabilitation that can continue for decades after their injury.

The management of burns is complex and multifaceted; however, high-quality burns care starts with first aid immediately after injury [4]. First aid is defined by the International Liaison Committee on Resuscitation (ILCOR) as “the helping behaviours and initial care provided for an acute illness or injury” [6]. In the case of burns, studies have demonstrated that the early application of appropriate first aid improves patient outcomes following injury [4,7-11]. Improved patient outcomes associated with cool water first aid treatment include a reduction in burn depth [8,10,12], faster re-epithelialization [10,12,13], lower rates of grafting [8,14-16] or less body surface area being grafted [10], shorter hospital length of stay [16-18], and significantly decreased rates of intensive care unit admission [16]. However, there is evidence to suggest that the public has a poor understanding of burns first aid and that there is often inconsistencies between the care recommendations in different guidelines [2,4,5,19,20]. Poor quality information available on the information and smartphone applications may contribute to the general public’s lack of understanding appropriate first aid for burns [21].

First aid guidelines provide an important reference for bystanders, first aid providers, and medical professionals to guide them in the effective provision of safe, effective, and timely first aid care. In Australia and New Zealand, the Australian and New Zealand Committee on Resuscitation (ANZCOR) provides first aid guidelines to guide care throughout the region. However, there are also a range of other reputable sources offering their own burns first aid guidelines which may be accessed by those seeking information. As such, it is imperative that there is minimal variation between national and international guidelines to reduce confusion and to ensure that recommendations are supported by best available evidence. With the ANZCOR first aid guidelines for burns last updated in 2016, this study aimed to conduct a rapid review of current first aid guidelines to identify any critical variation as well as identify any recent major changes in the literature that would warrant a significant change to current recommendations.

## Review

Rapid reviews are a form of evidence synthesis well-suited to answering focused research questions or assessing the current knowledge base surrounding a policy or practice. As they are conducted in a short period of time, they employ a simplified review process to produce information in a timely manner [22,23]. The search process comprised the following three phases: 1) a search for current international and Australian/New Zealand first aid guidelines for burns; 2) a search for burns first aid systematic reviews and meta-analyses published in the last six years (time since last ANZCOR burns first aid guidelines update); and 3) a search for current systematic reviews and clinical trials underway. Searches were conducted during the period of November to December 2020.

Guideline search

In order to identify major international and Australian/New Zealand guidelines, we searched known guideline databases, including the Australian National Health and Medical Research Council guidelines, Canadian Medical Association Infobase of Clinical Practice Guidelines, National Institute for Clinical Excellence, New Zealand Guidelines Group, ILCOR’s affiliated member databases, the WHO, and the Scottish Intercollegiate Guidelines Network. This was followed by an internet search using Google to identify other prominent first aid guidelines for burns.

Only guidelines written in English that primarily covered the first aid management of burns were included. Guidelines that covered advanced burns management within a specialist setting (e.g., a hospital or burns center) were excluded, as they do not fall within the realm of first aid as defined by ILCOR. For guidelines that included both first aid and more specialized management, only recommendations that could reasonably be provided in a first aid setting with minimal specialist equipment or training were included in our guideline comparison. If multiple versions of a guideline were identified from the same source, the most recent iteration was selected, ensuring the inclusion of any evidence-based updates. For each identified guideline, the key recommendations were extracted and assessed.

New evidence search

We conducted a literature search to identify any systematic reviews or meta-analyses published in the last decade on the topic of burns first aid. A search was conducted of the Medline, Embase, and Cochrane Database of Systematic Reviews electronic database using the search strategy provided in Tables 1, 2. The search period spanned from 2015 through to November 2020 (the period since the last ANZCOR review) and included any systematic review or meta-analysis published on the topic of burns first aid. Again, this included any treatment or practice that could reasonably be applied in a first aid situation without specialist equipment or training. Studies investigating specialized burns care were excluded including hospital care or advanced paramedical care. For each study, the results and conclusions were extracted.

**Table 1:** Literature Search Terms and Results: Medline

#	Searches	Results
1	Burn/	44,804
2	(burn* or scald* or brand* or singe*).mp.	127,569
3	((heat* or burn* or thermal or flame* or combust* or incinerat* or cook* or fire*) adj injur*).mp.	13,925
4	Emergency Medicine/ or Emergency Treatment/ or Emergency Medical Services/	24,200
5	(Prehospital or pre-hospital or prehospital care or first aid or first-aid or first responder of first response or emergency or emergency management or emergency medicine or first assistance or first medical aid or initial care or initial treatment or first help or urgent care or emergency aid or dressing or cool or cooling).mp.	353,148
6	1 or 2 or 3	130,594
7	4 or 5	353,148
8	6 and 7	7,068
9	Limit 8 to ((meta analysis or “systematic review”) and last 6 years	57

**Table 2:** Literature Search Terms and Results: Embase

#	Searches	Results
1	Burn/ or scald/ or heat injury/ or thermal injury/	74,006
2	(burn* or scald* or brand* or singe*).mp.	221,040
3	((heat* or burn* or thermal or flame* or combust* or incinerat* or cook* or fire*) adj injur*).mp.	28,943
4	Emergency Medicine/ or Emergency Treatment/ or Emergency Health Service/ or Burn dressing/ or first aid/ or cooling/	190,825
5	(Prehospital or pre-hospital or prehospital care or first aid or first-aid or first responder or first response or emergency or emergency management or emergency medicine or first assistance or first medical aid or initial care or initial treatment or first help or urgent care or emergency aid or dressing or cool or cooling).mp.	714,744
6	1 or 2 or 3	232,316
7	4 or 5	714,744
8	6 and 7	17,279
9	Limit 8 to ((meta analysis or “systematic review”) and last 6 years	220

Search for systematic reviews and clinical trials currently underway

A search of both the PROSPERO and ClinicalTrials.gov databases was conducted [24,25]. The inclusion criteria comprised systematic reviews or clinical trials currently in progress or due for publication covering first aid for burns. Exclusions were studies investigating specialized hospital burn care or advanced paramedical care. To determine whether any of the identified studies were already published, we also conducted a search for each study on Medline and Google using key identifiers including study title, author, and identification number.

Data extraction

A standardized data extraction table was developed including the following variables: safety, initial approach, cooling of heat/flame injuries, dressings/coverings, when to refer for medical treatment, additional management, chemical burns, electrical burns, and radiation burns. Data were extracted separately for Australian/New Zealand guidelines and international guidelines. Author M.M. extracted all data, and author L.M.T. verified the extracted data.

Results

Guideline Comparison

A total of 18 guidelines were identified that met the selection criteria for first aid guidelines covering burns. Seven guidelines were identified in the Australia/New Zealand region from ANZCOR [26], the Victorian Adult Burns Service (VABS) [27], the New South Wales Agency for Clinical Innovation (NSW ACI) [28], the Australian and New Zealand Burn Association (ANZBA) [29], Kidsafe Australia [30], St John Ambulance Australia [31], and the Royal Children’s Hospital Melbourne [32]. Eleven international guidelines were identified that met the rapid review criteria. These included guidelines from the International Society for Burn Injuries (ISBI) [4], the European Resuscitation Council (ERC) [11,33], the American Heart Association (AHA) [6,34], the European Burns Association (EBA) [35], the British Burn Association (BBA) [36], the American Burn Association (ABA) [37], the Center for Disease Control and Prevention (CDC) [38], the South African Burn Society [39], the WHO [1], Wounds International [40], and the International Federation of Red Cross and Red Crescent Societies [41]. Table 3 outlines the key recommendations made by Australian and New Zealand guidelines on first aid practices for burns. Table 4 outlines the same key recommendations made by the remaining international burns guidelines.

**Table 3:** Summary of Australian and New Zealand Burns First Aid Guidelines nd refers to no date ANZBA, Australian and New Zealand Burn Association; ANZCOR, Australian and New Zealand Committee on Resuscitation; NSW ACI, New South Wales Agency for Clinical Innovation; RCH, Royal Children’s Hospital, Melbourne; VABS, Victorian Adult Burns Service

	ANZBA (2019)	ANZCOR (2016)	Kidsafe Australia (nd)	NSW ACI (2019)	RCH (2018)	St John Ambulance Australia (nd)	VABS (2020)
Safety							
Rescuers/bystanders	Yes	Yes		Yes			
Patient		Yes					
Initial approach							
Extinguish flames	Yes	Yes		Yes		Yes	Yes
Extricate patient	Yes	Yes		Yes			
Call an ambulance		Yes	Yes		Yes	Yes	
Cooling of Heat/Flame Injuries							
Cool burn with running water for (at least) 20 minutes	Yes	Yes	Yes	Yes	Yes	Yes	Yes
Alternative recommendations if cool running water isn’t available	Yes						
Avoid use of ice or hypothermia	Yes	Yes	Yes	Yes	Yes	Yes	Yes
Lists other products to avoid using to cool burn	Yes			Yes			
Dressing/Coverings							
Remove clothing etc.	Yes	Yes	Yes	Yes	Yes	Yes	Yes
Cover with non-stick dressing, e.g., cling film	Yes	Yes	Yes	Yes	Yes	Yes	Yes
Keep patient warm	Yes	Yes					
Lists other products to avoid using to dress or cover burn	Yes	Yes	Yes		Yes		Yes
When to Refer to Medical Treatment	Yes	Yes	Yes	Yes	Yes	Yes	Yes
Additional Management							
Assess airway and breathing		Yes					
Check for other injuries		Yes					
Avoid hypothermia				Yes			
Administer oxygen if required		Yes					
Elevate burnt areas		Yes		Yes			
Chemical Burns		Yes		Yes			Yes
Electrical Burns		Yes					Yes
Radiation Burns		Yes					

**Table 4:** Summary of International Burns First Aid Guidelines nd refers to no date ABA, American Burn Association; AHA, American Heart Association; BBA, British Burn Association; CDC, Centre for Disease Control and Prevention; EBA, European Burns Association; ERC, European Resuscitation Council; IFRCRCS, International Federation of Red Cross and Red Crescent Societies; ISBI, International Society for Burn Injuries; SABS, South African Burn Society; WHO, World Health Organization; WI, Wounds International

	ABA (2017)	AHA (2015)	BBA (2018)	CDC (nd)	EBA (2017)	ERC (2015)	IFRCRCS (2011)	ISBI (2018)	SABS (2020)	WHO (2018)	WI (2014)
Safety											
Rescuers/bystanders							Yes	Yes		Yes	Yes
Patient								Yes			
Initial approach											
Extinguish flames			Yes	Yes				Yes	Yes	Yes	Yes
Extricate patient			Yes					Yes			
Call an ambulance							Yes				
Cooling of Heat/Flame Injuries											
Cool burn with running water for (at least) 20 minutes			Yes		Yes			Yes	Yes	Yes	Yes
Alternative recommendations if cool running water isn’t available		Yes	Yes					Yes			Yes
Avoid use of ice or hypothermia	Yes	Yes	Yes		Yes	Yes	Yes	Yes	Yes	Yes	Yes
Lists other products to avoid using to cool burn											
Dressing/Coverings											
Remove clothing etc.	Yes		Yes	Yes	Yes			Yes	Yes	Yes	Yes
Cover with non-stick dressing, e.g., cling film	Yes	Yes	Yes	Yes	Yes	Yes		Yes	Yes	Yes	Yes
Keep patient warm			Yes								
Lists other products to avoid using to dress or cover burn	Yes	Yes	Yes	Yes				Yes	Yes	Yes	Yes
When to Refer to Medical Treatment	Yes	Yes		Yes				Yes		Yes	Yes
Additional Management											
Assess airway and breathing											
Check for other injuries											Yes
Avoid hypothermia			Yes	Yes	Yes			Yes			
Administer oxygen if required								Yes			
Elevate burnt areas			Yes	Yes				Yes	Yes		
Chemical Burns			Yes				Yes	Yes		Yes	Yes
Electrical Burns			Yes					Yes		Yes	Yes
Radiation Burns											

Safety

When considering safety while administering first aid, only seven of 18 guidelines made comment on general safety principles such as ensuring bystander and responder safety when treating burns patients [1,4,26,28,29,40,41]. Passing references were made regarding safety in other guidelines in the form of statements such as "...when safe to do so" [36]. However, no specific focus on safety was featured.

Initial Approach

Fourteen of 18 guidelines provided some guidance on the initial approach to managing a patient with a burn injury, in particular the management of a patient engulfed in flames. Ten guidelines recommended the use of the “stop, drop, and roll” method of extinguishing a patient currently on fire [1,4,26-29,31,36,38-40]. Two guidelines mentioned the use of non-flammable liquids as a means of extinguishing flames [4,36].

Cooling of Heat/Flame Injuries

With regard to cooling, there was general consensus among all guidelines on active cooling of the burn injury. The majority of guidelines (n = 11), including all of the Australia/New Zealand guidelines, recommended exposing burn injuries to cool running water for at least 20 minutes [1,4,26-32,35,36,39,40]. Four international guidelines recommended shorter periods of cooling of less than 20 minutes [6,11,33,34,37,38], while two international guidelines did not quantify a recommended burn cooling duration [1,40]. Five guidelines recommended the use of alternating cool compresses in the event that fresh running water is in limited supply [6,29,34,36,38,40], and two guidelines suggested the use of hydrogels as an alternative to cool water [4,40]. In contrast, one guideline specifically stated that hydrogel dressings should not be used to cool the burn [28]. Thirteen of the 18 guidelines specified against the use of ice in cooling burn wounds [1,6,26-32,34,36,37,39-41]. There was general consensus among guidelines that special care must be taken when cooling burn wounds to not overcool the patient to avoid hypothermia, especially for large burns, and for burns in children and the elderly [1,4,6,11,33-37,39,40].

Dressings and Coverings

A majority of guidelines recommended the removal of burned, wet, or contaminated clothing from the patient [1,4,26-32,35-40]. A majority of guidelines also recommended the removal of any jewelry or other potentially constrictive garments [4,26-32,35-40]. Six guidelines recommend leaving any molten/adherent clothing in place if still attached to the skin [26,30-32,35,36,38,39]. There was no consensus among guidelines as to whether garments should be removed before or after cooling. A general consensus existed among guidelines that, after cooling, burn wounds should be covered in either a non-adherent, dry dressing [6,11,26,27,31,33-39], a clean cloth or sheet [1,4,29,30,36-38,40], or plastic wrap (cling film) [4,11,26-29,31-33,35,36,39,40]. One guideline specifically noted that cling film should not be applied to faces or facial burns [36]. However, most guidelines suggested only one or two dressing options, with only one guideline suggesting all three dressings as an option [36]. A general consensus existed among guidelines to avoid the use of ointments as first aid for burns wounds [1,4,6,26,27,29-32,34,37-40]. This includes the use of butter, creams, salves, and other home remedies such as toothpaste or porridge.

When to Refer to Medical Treatment

Significant variation between guidelines existed on when a burn should be referred for medical evaluation, and some guidelines provided no referral criteria. Some guidelines suggested that any burn greater than a certain total body surface area should be referred for medical assessment in both adults and children [1,27-29,37,40], while others use a specific sizing guide (usually based on diameter) [30-32,37], or instead used non-specific language such as “large” or “significant” [4,6,29,31,34,38]. Several guidelines mentioned that burns involving functional areas such as the face, neck, hands, genitals, or over joints should be evaluated by a medical professional [1,6,27,29,30,32,34,37,40]. Two guidelines mentioned that chemical burns involving the eye should be referred to an ophthalmologist for immediate evaluation [4,26].

Additional Management

One guideline from Australia and New Zealand advised rescuers to check the patient’s airway and how adequately they could breathe [26], while two guidelines recommended oxygen administration to patients with inhalation injuries [4,26]. Seven guidelines (five of which were from an international association or organization) recommended elevating limbs and/or the burned area to prevent edema [4,26,28,36,38,39].

Chemical, Electrical, and Radiation Burns

Eight guidelines made mention of first aid for chemical burns [1,4,26-28,36,40,41]. Of these, there was general consensus on prolonged irrigation of the wound with water [4,26-28,36,40,41]. There was differing advice provided on the ideal temperature of the water, with some guidelines suggesting cool water, another suggesting room temperature, and many not specifying. It is important to note that alternative first aid approaches to chemical burns have been suggested in the literature besides those presented in the guidelines we examined [42-44]. One guideline suggested sterile isotonic or amphoteric solutions as an alternative to water for chemical burns [36]. Some variation existed between the guidelines on the duration of irrigation, with ranges between 20 minutes to two hours being suggested, or until pain subsides. There was general consensus among all guidelines that mentioned chemical burns that eye involvement in a chemical burn should warrant prolonged eye irrigation with water or normal saline [4,26-28,36,40,41].

Eight guidelines made mention of first aid for electrical burns [1,4,6,26,27,34,36,40,41]. There was a general consensus among guidelines on the overall management of electrical burns including isolating/turning off the electrical source, administering cardiopulmonary resuscitation (CPR) as appropriate, and cooling burns with running tap water for 20 minutes if appropriate. One guideline specified that irrigation with cool running water was not appropriate due to the depth of electrical burns [40]. Only one guideline covered the treatment of radiation burns [26]. This guideline suggested covering the burns with a clean, dry dressing.

New Evidence

The literature search yielded 57 results from OVID Medline, 220 results from Embase, and 23 results from the Cochrane Database of Systematic Reviews. After removing duplicate studies, three studies met the inclusion criteria; all were systematic reviews [45-47]. One systematic review by Goodwin et al. investigated the use of hydrogel dressings in the prehospital setting but did not identify any suitable studies that met their inclusion criteria [46]. Another systematic review by Barqouni et al. investigated the management of phosphorous burns. This review reported no evidence from two non-randomized studies to support the use of copper sulfate [45]. Indeed, the small amount of available evidence suggested that it may be harmful. Barqouni et al. concluded that phosphorous burns be treated by removing the patient’s clothing and continuously irrigating the burn with a cold solution to remove phosphorous particles. A final systematic review by Harshman et al. looked at aspects of pre-burn center care of burn wounds. While the majority of findings discussed by Harshman et al. were more relevant for trained healthcare professionals with specialist equipment, one relevant finding was that burn wounds were not consistently covered with a dry dressing or clean sheet in the prehospital or emergency setting [47].

Systematic Reviews and Clinical Trials Currently Underway

The search of the PROSPERO database yielded two systematic reviews currently underway. One aimed to review all aspects of prehospital management of severe burns; however, the authors have not provided enough detail to assess the usefulness of their study to first aid [48]. The other aimed to determine if the application of 20 minutes of cool running water within three hours of thermal burn injury was more effective than alternative remedies [49]. The search of ClinicalTrials.gov did not yield any relevant results for upcoming studies involving burns first aid care.

Discussion

Our results indicate that there is key variation between guidelines on first aid for burn injuries. While there is a general consensus among guidelines on the use of cool running water for cooling of burns, there is still some variation in the duration of cooling recommended by some guidelines. This variation could be explained by the differing evidence available at the date these guidelines were last updated as well as the lack of or low level of evidence supporting some treatments. Due to the crucial nature of early and effective cooling in the management of burn wounds, consistency needs to be promoted across first aid guidelines to ensure that wound cooling recommendations are in line with the best available evidence.

The most recent ILCOR evidence review on cooling burns was conducted in 2015 and resulted in a broad treatment recommendation that first aid providers actively cool thermal burns (low-quality evidence) [33]. This recommendation was based on limited evidence, from one randomized controlled trial and four observational studies, and could only suggest cooling for a minimum of 10 minutes with no clear evidence found for temperature or method of cooling. Our literature search of recent systematic reviews and meta-analyses has not revealed any new evidence on this subject, although additional cohort studies have since been published [9,10]. This suggests the need for further research in these areas as well as a consensus on best practice (by consensus of expert opinion) until more research has been published. However, it should be noted that ILCOR released a draft of an updated Consensus on Science with Treatment Recommendations (CoSTR) systematic review that investigated the duration of cooling with water for thermal burns as a first aid intervention [50]. The CoSTR stated that there was insufficient evidence to specify duration of cooling. While waiting for this research, it would help ordinary first aiders to have a consensus in the guidelines they consult, whether by the Delphi or other process.

Dressings/wound coverings were another area in the guidelines where variation existed. There were three main recommendations made across each of the guidelines: dry dressings, a clean cloth or sheet, and plastic cling film (with one guideline noting that cling film should not be applied to the face or facial burns). However, most guidelines only recommended one or two of these options, with only one guideline recommending all three as potential dressing options. This is an area where consistency across guidelines should be encouraged in order to provide as many dressing options as possible across different first aid settings.

An area where significant variation existed among guidelines was advice as to when to seek medical evaluation for burns. While there was a general consensus across guidelines that burns to special areas of the body (e.g., face, hands, and genitals) should be seen by a burn specialist, there was less consistency among guidelines on other factors (e.g., size and severity of burn, comorbidities, or other risk factors) that would warrant referral to a medical practitioner. Some guidelines appeared to take a very cautious approach to their referral criteria by suggesting that any concerning or severe burns should be seen by a medical practitioner. However, in this case, many failed to define what made a burn concerning or serious. Other guidelines appeared to have stricter referral criteria that would preclude a number of burn injuries that would usually necessitate medical evaluation and intervention. It is worth noting that many bystanders and first responders may not be able to accurately differentiate between a superficial, partial-thickness, and full-thickness burn. Similarly, many may not know what constitutes a “large” or “significant” burn, which highlights a need for clarification within these guidelines. This variation between guidelines highlights a need for a more consistent evidence-based international referral criteria standard to be developed. Given that a number of guidelines investigated in this report are from member organizations of ILCOR, it is important to recognize that a concerted effort has been made to standardize the first aid for burns by ILCOR. However, there is significant variation in areas not covered by the Continuous Evidence Evaluation and CoSTR publications, namely methods of determining burn area and which burns need further medical care. While these could be considered teaching points, the international variation suggests the need for further guidance on these points.

Other key areas where guideline variability existed include general safety principles and the initial approach to a burning patient. While both are simple concepts, two-thirds of guidelines mentioned the importance of ensuring first responder and bystander safety when treating a burns patient. Similarly, only two-thirds of guidelines mentioned the “stop, drop, and roll” method of extinguishing an actively burning patient, and only two guidelines mentioned extinguishing flames with a non-flammable liquid such as water. While these interventions are usually assumed to be “common sense,” guidelines should be encouraged to make mention of them in order to provide a holistic approach to the management of a patient with burns. This is especially pertinent as there is a body of evidence showing that the general public has a limited understanding of burns first aid, and therefore every effort should be made to improve this.

There were a number of areas where a general consensus existed among the guidelines. This included avoiding ice as a cooling method, as well as avoiding salves, creams, and other home remedies as cooling methods. There was also consensus among the guidelines that included the management of chemical and electrical burns. While there was a general consensus among guidelines that contaminated clothing and jewelry should be removed from the patient, some confusion still remains about whether garments should be removed before or after cooling and whether molten/adherent clothing should be left in place or removed. Further clarity on this point needs to be achieved.

There was some variation between the Australian/New Zealand guidelines and the international guidelines. All of the Australian/New Zealand guidelines recommended 20 minutes of cooling, and all made recommendations about when to refer a burn for medical evaluation in some form. Many of the international guidelines had variation in cooling duration, and many did not make any mention of when to refer a burn for medical evaluation.

The literature review we conducted did not provide any additional evidence to support or refute current recommendations for prehospital management of burns. Our search of PROSPERO and ClinicalTrials.gov yielded only two potential studies that met our inclusion criteria, which was of marginal quality. This suggests that there is a lack of recent and high-quality systematic reviews or meta-analyses in this area and that further research into first aid management of burns should be conducted. However, the search of PROSPERO did reveal additional systematic reviews outside our inclusion criteria that were either underway or complete (but not published). While there are potential barriers to developing high-level evidence in the prehospital setting, given the critical nature of those first stages of burn management, further research should be conducted to guide future guideline recommendations.

Limitations

It is important to acknowledge the potential limitations of our review. While every effort has been made to identify suitable guidelines for inclusion in our study, there is potential for some guidelines to have been overlooked. Additionally, while we aimed to conduct a methodological search for systematic reviews and meta-analyses, due to the nature of a rapid review, the search was not systematic in its approach, and thus it is possible that we have missed suitable studies published during our search period that warrant discussion. Finally, it should be acknowledged that some guidelines in our review may currently be under review or shortly due for an update. This may alter the variability between them and other guidelines; however, our rapid review suggests that there is unlikely to be any significant change to the treatments we evaluated.

## Conclusions

Our rapid review identified key variation between first aid guidelines for burns that would benefit from the development of an international consensus on management. There was variation between the Australian/New Zealand guidelines and international guidelines concerning cooling duration and criteria for when to refer for medical evaluation. We identified no new significant evidence that would alter guideline recommendations and did not identify any upcoming reviews or clinical trials on this subject.
